# Continuous response to maintenance fuzuloparib for germline BRCA2- mutated metastatic pancreatic adenocarcinoma: a case report and literature review

**DOI:** 10.3389/fphar.2025.1656670

**Published:** 2025-09-09

**Authors:** Chenyan Zhang, Pei Zhang, Ke Cheng, Dan Cao

**Affiliations:** Division of Abdominal Tumor, Department of Medical Oncology, Cancer Center and State Key Laboratory of Biological Therapy, West China Hospital, Sichuan University, Chengdu, Sichuan, China

**Keywords:** gBRCA-mutation, pancreatic cancer, PARP inhibitor, platinum-based chemotherapy, Metastasis

## Abstract

Pancreatic adenocarcinoma with germline BRCA mutations (gBRCAm) represents a distinct molecular subtype with enhanced sensitivity to platinum-based chemotherapy and poly (ADP-ribose) polymerase inhibitors (PARP inhibitors). Fuzuloparib is a novel, potent, and orally bioavailable PARP inhibitor. Despite showing improved efficacy and a more favorable safety profile compared to olaparib in preclinical studies, clinical evidence for its application in pancreatic adenocarcinoma harboring gBRCAm remains limited. We report here a 33-year-old Asian woman with extensively metastatic pancreatic adenocarcinoma harboring a germline BRCA2 nonsense mutation who a durable response to fuzuloparib after NALIRIFOX chemotherapy. Her progression-free survival exceeded 15 months with ongoing fuzuloparib maintenance therapy for over 7 months. This case underscores the important role of biomarker-directed therapy in pancreatic adenocarcinoma and fuzuloparib may represent a potential PARP inhibitor option for maintenance treatment in pancreatic adenocarcinoma with gBRCAm. However, large-scale randomized controlled trials are needed to validate these results.

## Introduction

Pancreatic adenocarcinoma is a lethal condition, which has currently become the third leading cause of cancer-related deaths in the United States (National Cancer Institute, 2025). Despite advancements in chemotherapy, the overall prognosis for patients with metastatic pancreatic adenocarcinoma remains unfavorable due to its diverse molecular subgroups and unique biological characteristics (Von Hoff et al., 2013; Conroy et al., 2011; Park et al., 2021; Wainberg et al., 2023). Hence, understanding the molecular basis of different genomic mutation profiles is critical for implementing biomarker-directed therapy.

BRCA1 and BRCA2 are crucial proteins involved in homologous recombination repair (HRR), loss of function of these genes results in homologous recombination deficiency (HRD) (Tutt and Ashworth, 2002). Approximately 4%–7% of pancreatic adenocarcinoma harbor germline BRCA mutations (gBRCAm) (Holter et al., 2015). This genetic characteristic confers enhanced sensitivity to platinum-based agents and poly (adenosine diphosphate–ribose) polymerase (PARP) inhibitors in pancreatic adenocarcinoma patients (Ghiorzo, 2014; O'Reilly et al., 2020; Kindler et al., 2022). The landmark POLO trial established that olaparib represents the standard maintenance therapy in patients with metastatic pancreatic adenocarcinoma harboring gBRCAm who demonstrated sensitivity to platinum-based chemotherapy (Golan et al., 2019).

Fuzuloparib is a novel, potent, and orally bioavailable PARP inhibitor that has demonstrated superior *in vivo* efficacy and improved safety profile compared to olaparib in preclinical studies (Li et al., 2020a). In the FZOCUS-2 study, fuzuloparib as maintenance therapy achieved statistically significant survival improvement with tolerable adverse events in patients with platinum-sensitive recurrent ovarian cancer (Li et al., 2022). However, clinical data regarding fuzuloparib as maintenance therapy in pancreatic adenocarcinoma remains limited.

Here, we present a remarkable case of a patient with extensively metastatic pancreatic adenocarcinoma harboring gBRCAm, who demonstrated sensitive to NALIRIFOX chemotherapy. Notably, the tumor lesions exhibited continued modest regression during subsequent maintenance fuzuloparib.

## Case presentation

**FIGURE 1:**
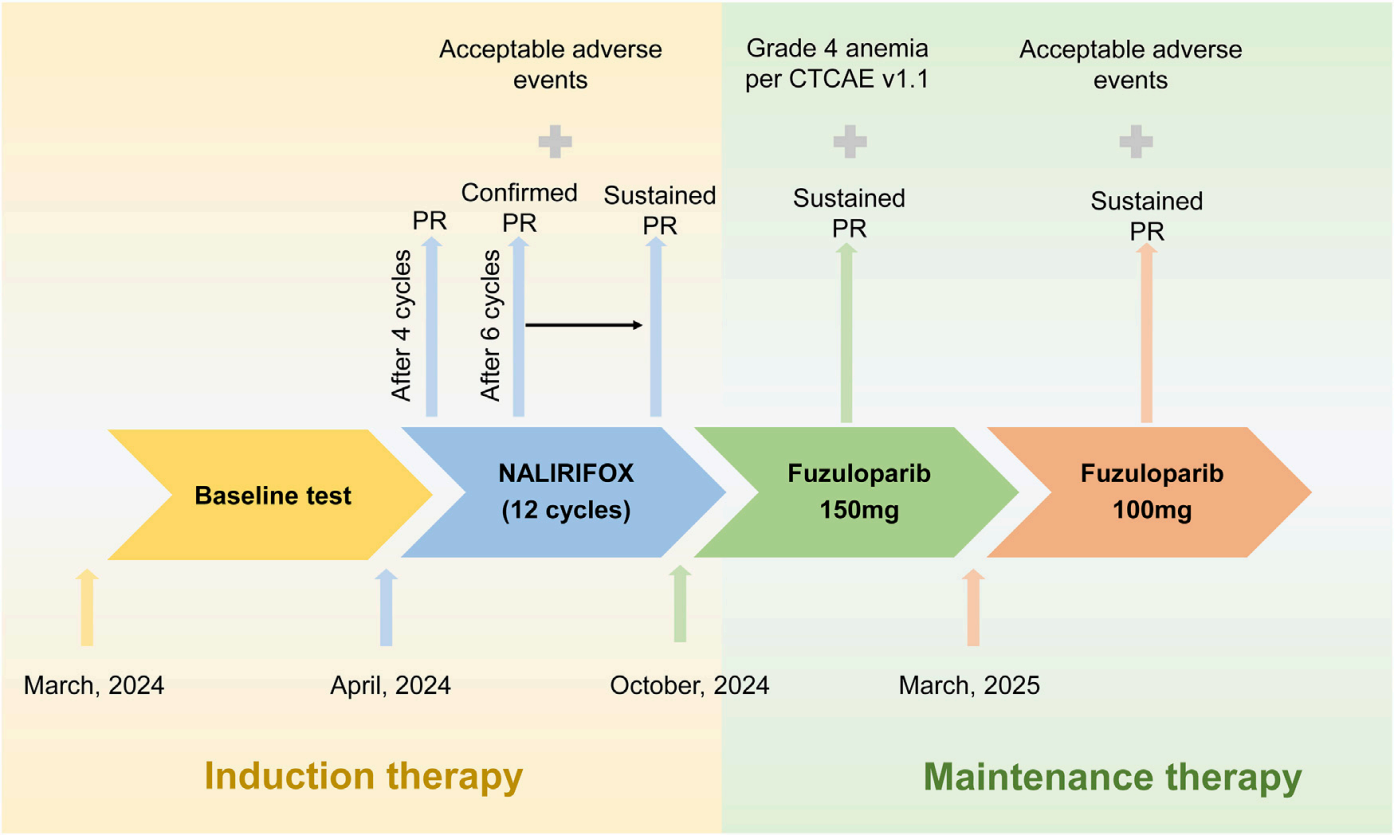
Treatment timeline and clinical response in a patient with gBRCA2-mutated metastatic pancreatic adenocarcinoma. The schematic timeline illustrates the sequential treatment phases from March 2024 to April 2025. Following baseline assessment in March 2024, the patient received 12 cycles of NALIRIFOX induction therapy (April to October 2024), achieving partial response (PR) after four cycles with sustained PR throughout induction. Based on stable disease with continued tumor shrinkage, adequate performance status (ECOG 0), and patient preference for oral maintenance therapy, fuzuloparib maintenance therapy was initiated at 150 mg twice daily in November 2024, maintaining continued tumor response. Due to grade 4 hematologic toxicity in March 2025, fuzuloparib was dose-reduced to 100 mg twice daily with resolution of adverse events and sustained PR. The arrows indicate treatment progression, and crosses denote adverse events. PR, partial response; CTCAE, Common Terminology Criteria for Adverse Events.

**FIGURE 2:**
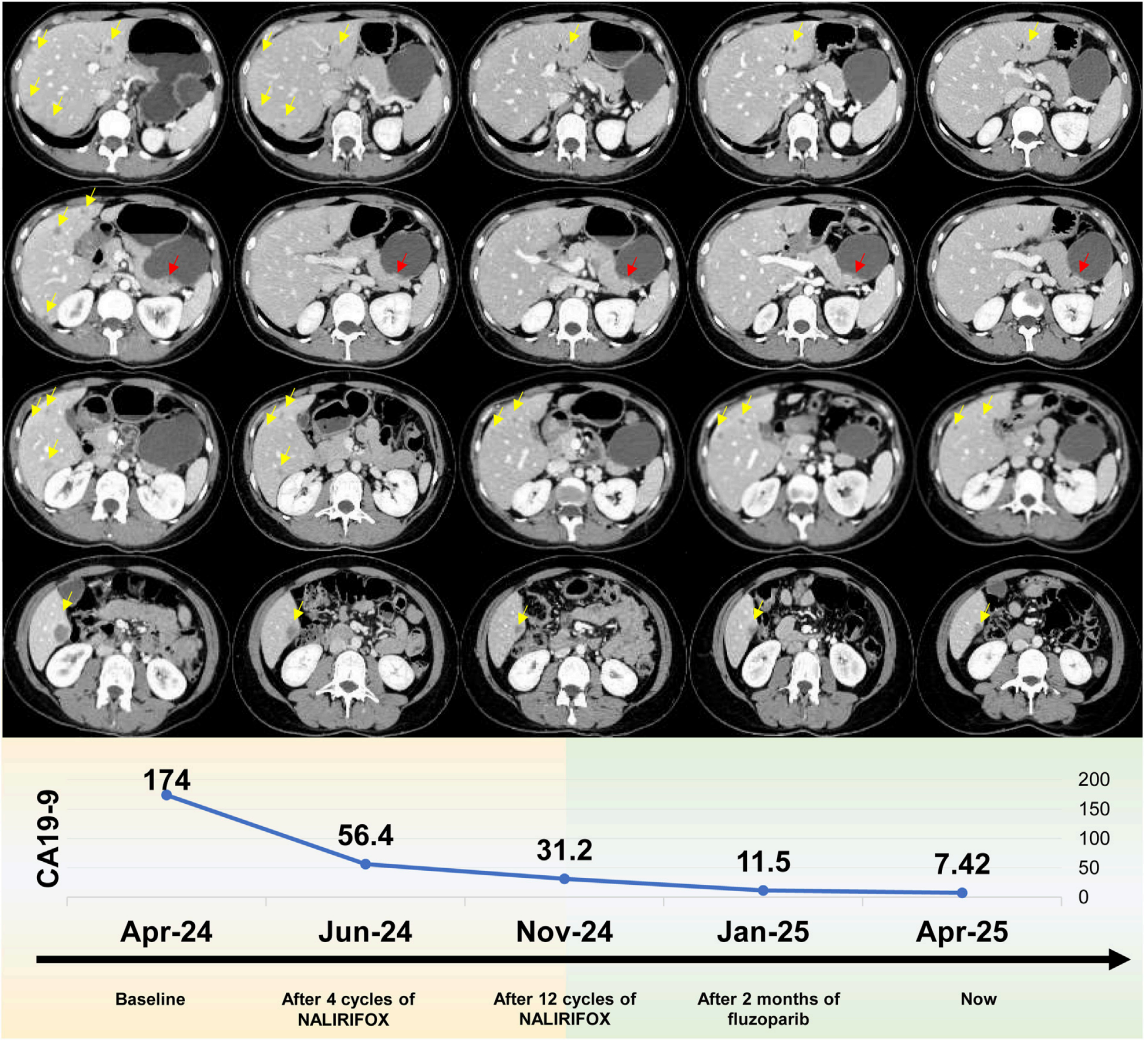
Radiological progression and tumor marker response during treatment. Upper panel: Serial computed tomography (CT) imaging. Representative axial CT scans demonstrating dynamic tumor response from baseline through maintenance therapy. Yellow arrows indicate hepatic metastases, while red arrows denote the pancreatic tail primary tumor. Sequential imaging reveals progressive tumor regression from baseline (leftmost column) continuing through fuzuloparib maintenance therapy (rightmost column). The imaging timeline encompasses: baseline evaluation (March 2024), post-4 cycles of NALIRIFOX (June 2024), post-12 cycles of NALIRIFOX (November 2024), following 2 months of fuzuloparib maintenance (January 2025), and current assessment (April 2025). Lower panel: CA19-9 tumor marker kinetics. The graph demonstrates the remarkable decline in serum CA19-9 concentrations (U/mL) from an elevated baseline of 174 U/mL to current normalized levels of 7.42 U/mL throughout the treatment continuum. Temporal measurements correspond to baseline assessment, post-4 cycles of NALIRIFOX, post-12 cycles of NALIRIFOX, following 2 months of fuzuloparib maintenance, and current evaluation, illustrating sustained biochemical response that closely parallels the radiological findings.

A 33-year-old asymptomatic Asian woman was found to have masses in pancreatic tail and liver on routine screening, with ECOG performance status 0. Positron emission tomography-computed tomography (PET-CT) (1 March 2024), revealed a solid-cystic mass in the pancreatic tail and multiple hypermetabolic hepatic lesions (Figure 1; Supplementary Figure A1). Serum CA19-9 level was elevated at 186 U/mL (normal range: <37 U/mL) (Figure 2). Liver biopsy confirmed pancreatic adenocarcinoma with the following immunohistochemical (IHC) markers: CK7 (+), CK8/18 (+), CD56 (−), CK5/6 (−), CR (−), WT1 (−), p63 (−), CDX2 (−), CK20 (−), HepPar-1 (−), and Ki-67 (20%). Comprehensive next-generation sequencing (NGS) analysis revealed a pathogenic germline BRCA2 nonsense mutation (Table 1). Notably, the patient reported no relevant personal history of cancer or family history of BRCA-associated malignancies.

**TABLE 1:** Patient next-generation sequencing results.

Gene	Transcription book	Base change	Amino acid change	Functional area	Mutation frequency (%)
BRCA2	NM_000059.3	c.3109C>T	p.Q103 7*	EX11	germline mutation
TSC2	NM_000548.3	c.2838 8_2854delCCCGAT AGTCTGAGGATAG CCAGAC	.	IVS25-EX26	3.0
FLT4	NM_182925.4	c.3432–17_3432 1delCCCACGTGATCCTGCAG	.	IVS25	5.5
TGFBR2	NM_001024847.2	c.17_40delTCAGGGGCCTGTGGCC GCTGCACAinsCATGGG	p.L6_I14delinsPWV	EX1	2.4

Given the gBRCAm, first-line treatment with NALIRIFOX was initiated on 17 April 2024 (Figure 1). After four cycles, CT showed a deep and rapid partial response (PR) per Response Evaluation Criteria in Solid Tumors version 1.1 (RECIST v1.1), with CA19-9 dropping to 56.4 U/mL (Figure 2). The impressive response continued with ongoing treatment. (Figures 1, 2).

After 12 cycles of NALIRIFOX induction therapy, the patient achieved significant treatment response with CA19-9 reduction from 174 to 31.2 U/mL and sustained PR confirmed by CT imaging per RECIST v1.1 (Figure 2). Given the stable disease with continued tumor shrinkage, excellent performance status (ECOG 0), patient preference for oral maintenance therapy, and cumulative oxaliplatin-induced peripheral neuropathy following multiple cycles of platinum-based chemotherapy, maintenance therapy with fuzuloparib 150 mg twice daily was initiated in November 2024, resulting in continued PR and CA19-9 normalization to 11.5 U/mL (Figures 1, 2). This dose of fuzuloparib was selected based on existing clinical trial data demonstrating optimal efficacy and tolerability profiles (Li et al., 2020a; Li et al., 2022; Wei et al., 2024). However, severe hematologic toxicities developed in March 2025, including grade 4 anemia (hemoglobin 49 g/L; normal range: 110–150 g/L), grade 3 leukopenia (white blood cell 2.91 × 10^9^/L; normal range: 4.0–10.0 × 10^9^/L), grade 3 thrombocytopenia (platelets 83 × 10^9^/L; normal range: 100–300 × 10^9^/L), and grade 3 neutropenia (neutrophils 1.5 × 10^9^/L; normal range: 2.0–7.0 × 10^9^/L) according to Common Terminology Criteria for Adverse Events (CTCAE v5.0) criteria. Following supportive care including blood transfusion and granulocyte colony-stimulating factor (G-CSF) administration, along with fuzuloparib dose reduction to 100 mg twice daily, the patient continues on maintenance therapy with resolved toxicities (Figure 1), demonstrating grade 1 anemia (hemoglobin 99 g/L) with normalized white blood cell and platelet counts. Notably, following fuzuloparib dose reduction, the pancreatic and hepatic lesions exhibited further modest regression, and serum CA19-9 levels continued to decline (Figure 2). As of 21 June 2025, the patient has achieved progression-free survival (PFS) exceeding 15 months, with ongoing fuzuloparib maintenance therapy for over 7 months.

## Discussion

We present a remarkable case of a female patient with metastatic pancreatic adenocarcinoma harboring gBRCAm. Following 12 cycles of NALIRIFOX induction therapy, she achieved PR. Continued modest tumor regression was observed during subsequent fuzuloparib maintenance therapy.

GBRCAm provides unique therapeutic opportunities for pancreatic adenocarcinoma (Golan et al., 2014). Platinum-based chemotherapies exert their cytotoxic effect by binding directly to DNA, causing crosslinking of DNA strands and inducing DNA double-strand breaks, which cannot be effectively repaired in pancreatic adenocarcinoma with pathogenic or likely pathogenic gBRCA mutations (Farmer et al., 2005; Li et al., 2020b). This phenomenon results in preferential tumor cell death and confers superior survival outcomes to patients with gBRCA variants treated with platinum-based chemotherapy, which was validated in many clinical trials (O'Reilly et al., 2020; Golan et al., 2014; Wattenberg et al., 2020). In our case, considering the patient’s germline BRCA2 nonsense mutation, we prioritized NALIRIFOX or FOLFIRINOX as first-line therapy. Based on the favorable survival outcomes demonstrated by NALIRIFOX in the NAPOLI-3 study, NALIRIFOX was ultimately selected (Wainberg et al., 2023). Moreover, the patient demonstrated excellent tumor regression and survival following 12 cycles of NALIRIFOX induction chemotherapy, which underscores the important role of biomarker-directed therapy in first-line chemotherapy selection for pancreatic cancer.

PARP inhibitors represent a targeted therapeutic approach for pancreatic adenocarcinoma with gBRCAm. PARP inhibitors exploit homologous recombination deficiency by inhibiting base excision repair and trapping PARP enzymes, ultimately leading to replication fork collapse and inducing lethal double-strand DNA breaks specifically in BRCA-deficient tumors (Das et al., 2024). Although the POLO study demonstrated survival benefits of olaparib as maintenance therapy in pancreatic cancer patients harboring gBRCAm, olaparib also exhibited significant hematological and gastrointestinal toxicities (Golan et al., 2019). Fuzuloparib, as a novel PARP inhibitor, demonstrated superior *in vivo* efficacy and reduced toxicity compared to olaparib in preclinical studies and clinical studies (Li et al., 2020a; Topatana et al., 2020). However, clinical data for fuzuloparib in gastrointestinal malignancies remains limited. Currently, only a phase Ib study conducted in 2023 explored the efficacy of fuzuloparib combined with chemotherapy in pancreatic cancer, demonstrating that fuzuloparib maintenance therapy following FOLFIRINOX in advanced pancreatic adenocarcinoma showed favorable efficacy and manageable tolerability (Wei et al., 2024). Additional studies have reported that fuzuloparib 150 mg twice daily as maintenance therapy demonstrated favorable efficacy and tolerability profiles in lung and ovarian cancers (Li et al., 2022; Li et al., 2023). In this case, considering the patient’s economic constraints, we selected maintenance fuzuloparib as maintenance therapy in this case. Although the patient developed grade 4 myelosuppression after 2 months of fuzuloparib 150 mg twice daily, sustained modest tumor regression with tolerant adverse events was observed following dose reduction. These findings suggest that fuzuloparib may represent a potential PARP inhibitor option for maintenance treatment in pancreatic adenocarcinoma with gBRCAm; however, large-scale randomized controlled trials are needed to validate these results.

With the emergence of targeted therapies for KRAS mutations, DNA damage repair deficiencies, and mismatch repair pathways, targeted treatment has become a research hotspot in pancreatic cancer (Li et al., 2024). In this case, the patient harbored additional mutations in TSC2, FLT4, and TGFBR2 genes. Research on targeted therapies for FLT4 and TGFBR2 mutations remains limited in pancreatic cancer. TSC2 functions as a key negative regulator of mTORC1 signaling (Manning and Cantley, 2003). However, the efficacy of mTOR inhibitors such as everolimus and ridaforolimus in pancreatic cancer remains controversial (Mortazavi et al., 2022). Some studies have demonstrated that in TSC2-mutated cancer both combination therapy with PD-1 and CTLA-4 antibodies, as well as monotherapy, can enhance CD8^+^ T cell infiltration in TSC2-deficient human tumors, with the level of infiltration correlating with treatment response (Liu et al., 2018). In 2021, a case report documented successful clinical outcomes using this approach in pancreatic cancer (Wang et al., 2014). However, further clinical research is currently lacking to support this perspective. These genetic alterations may provide potential avenues for future targeted and immunotherapy strategies in pancreatic cancer patients.

## Conclusion

This study presents a remarkable case of gBRCAm metastatic pancreatic adenocarcinoma that demonstrated a durable response to fuzuloparib after NALIRIFOX chemotherapy. This case further reinforces the important role of biomarker-directed therapy in pancreatic adenocarcinoma. Furthermore, it underscores that fuzuloparib may represent a potential PARP inhibitor option for maintenance treatment in pancreatic adenocarcinoma with gBRCAm; however, large-scale randomized controlled trials are needed to validate these results.

## Data Availability

The original contributions presented in the study are included in the article/Supplementary Material, further inquiries can be directed to the corresponding authors.

## References

[B1] Conroy T. Desseigne F. Ychou M. Bouché O. Guimbaud R. Bécouarn Y. (2011). FOLFIRINOX *versus* gemcitabine for metastatic pancreatic cancer. N. Engl. J. Med. 364 (19), 1817–1825. 10.1056/NEJMoa1011923 21561347

[B2] Das P. K. Matada G. S. P. Pal R. Maji L. Dhiwar P. S. Manjushree B. V. (2024). Poly (ADP-ribose) polymerase (PARP) inhibitors as anticancer agents: an outlook on clinical progress, synthetic strategies, biological activity, and structure-activity relationship. Eur. J. Med. Chem. 274, 116535. 10.1016/j.ejmech.2024.116535 38838546

[B3] Farmer H. McCabe N. Lord C. J. Tutt A. N. Johnson D. A. Richardson T. B. (2005). Targeting the DNA repair defect in BRCA mutant cells as a therapeutic strategy. Nature 434 (7035), 917–921. 10.1038/nature03445 15829967

[B4] Ghiorzo P. (2014). Genetic predisposition to pancreatic cancer. World J. gastroenterology 20 (31), 10778–10789. 10.3748/wjg.v20.i31.10778 25152581 PMC4138458

[B5] Golan T. Kanji Z. S. Epelbaum R. Devaud N. Dagan E. Holter S. (2014). Overall survival and clinical characteristics of pancreatic cancer in BRCA mutation carriers. Br. J. Cancer 111 (6), 1132–1138. 10.1038/bjc.2014.418 25072261 PMC4453851

[B6] Golan T. Hammel P. Reni M. Van Cutsem E. Macarulla T. Hall M. J. (2019). Maintenance olaparib for germline BRCA-mutated metastatic pancreatic cancer. N. Engl. J. Med. 381 (4), 317–327. 10.1056/NEJMoa1903387 31157963 PMC6810605

[B7] Holter S. Borgida A. Dodd A. Grant R. Semotiuk K. Hedley D. (2015). Germline BRCA mutations in a large clinic-based cohort of patients with pancreatic adenocarcinoma. J. Clin. Oncol. 33 (28), 3124–3129. 10.1200/jco.2014.59.7401 25940717

[B8] Kindler H. L. Hammel P. Reni M. Van Cutsem E. Macarulla T. Hall M. J. (2022). Overall survival results from the POLO trial: a phase III study of active maintenance olaparib *versus* placebo for germline BRCA-mutated metastatic pancreatic cancer. J. Clin. Oncol. 40 (34), 3929–3939. 10.1200/jco.21.01604 35834777 PMC10476841

[B9] Li H. Liu R. Shao B. Ran R. Song G. Wang K. (2020a). Phase I dose-escalation and expansion study of PARP inhibitor, fluzoparib (SHR3162), in patients with advanced solid tumors. Chin. J. Cancer Res. 32 (3), 370–382. 10.21147/j.issn.1000-9604.2020.03.08 32694901 PMC7369176

[B10] Li H. LaDuca H. Pesaran T. Chao E. C. Dolinsky J. S. Parsons M. (2020b). Classification of variants of uncertain significance in BRCA1 and BRCA2 using personal and family history of cancer from individuals in a large hereditary cancer multigene panel testing cohort. Genet. Med. 22 (4), 701–708. 10.1038/s41436-019-0729-1 31853058 PMC7118020

[B11] Li N. Zhang Y. Wang J. Zhu J. Wang L. Wu X. (2022). Fuzuloparib maintenance therapy in patients with platinum-sensitive, recurrent ovarian carcinoma (FZOCUS-2): a multicenter, randomized, double-blind, placebo-controlled, phase III trial. J. Clin. Oncol. 40 (22), 2436–2446. 10.1200/jco.21.01511 35404684

[B12] Li D. Huang Z. Zhong J. Lin L. Zhang G. Zhuang W. (2023). Efficacy and safety of fluzoparib combined with anlotinib in extensive stage small cell lung cancer after first-line platinum-based chemotherapy: a multi-center, single-arm prospective phase II clinical study (STAMP study). BMC cancer 23 (1), 753. 10.1186/s12885-023-11230-5 37580661 PMC10424452

[B13] Li B. Zhang Q. Castaneda C. Cook S. (2024). Targeted therapies in pancreatic cancer: a new era of precision medicine. Biomedicines 12 (10), 2175. 10.3390/biomedicines12102175 39457488 PMC11505516

[B14] Liu H. J. Lizotte P. H. Du H. Speranza M. C. Lam H. C. Vaughan S. (2018). TSC2-deficient tumors have evidence of T cell exhaustion and respond to anti-PD-1/anti-CTLA-4 immunotherapy. JCI insight 3 (8), e98674. 10.1172/jci.insight.98674 29669930 PMC5931128

[B15] Manning B. D. Cantley L. C. (2003). United at last: the Tuberous sclerosis complex gene products connect the phosphoinositide 3-kinase/Akt pathway to Mammalian target of rapamycin (mTOR) signalling. Biochem. Soc. Trans. 31 (Pt 3), 573–578. 10.1042/bst0310573 12773158

[B16] Mortazavi M. Moosavi F. Martini M. Giovannetti E. Firuzi O. (2022). Prospects of targeting PI3K/AKT/mTOR pathway in pancreatic cancer. Crit. Rev. Oncol. Hematol. 176, 103749. 10.1016/j.critrevonc.2022.103749 35728737

[B17] National Cancer Institute (2025). Surveillance, epidemiology and end results program. Available online at: https://seer.cancer.gov/ (Accessed July, 2025).

[B18] O'Reilly E. M. Lee J. W. Zalupski M. Capanu M. Park J. Golan T. (2020). Randomized, multicenter, phase II trial of gemcitabine and cisplatin with or without veliparib in patients with pancreas adenocarcinoma and a germline BRCA/PALB2 mutation. J. Clin. Oncol. 38 (13), 1378–1388. 10.1200/jco.19.02931 31976786 PMC7193749

[B19] Park W. Chawla A. O'Reilly E. M. (2021). Pancreatic cancer: a review. Jama 326 (9), 851–862. 10.1001/jama.2021.13027 34547082 PMC9363152

[B20] Topatana W. Juengpanich S. Li S. Cao J. Hu J. Lee J. (2020). Advances in synthetic lethality for cancer therapy: cellular mechanism and clinical translation. J. Hematol. Oncol. 13 (1), 118. 10.1186/s13045-020-00956-5 32883316 PMC7470446

[B21] Tutt A. Ashworth A. (2002). The relationship between the roles of BRCA genes in DNA repair and cancer predisposition. Trends Mol. Med. 8 (12), 571–576. 10.1016/s1471-4914(02)02434-6 12470990

[B22] Von Hoff D. D. Ervin T. Arena F. P. Chiorean E. G. Infante J. Moore M. (2013). Increased survival in pancreatic cancer with nab-paclitaxel plus gemcitabine. N. Engl. J. Med. 369 (18), 1691–1703. 10.1056/NEJMoa1304369 24131140 PMC4631139

[B23] Wainberg Z. A. Melisi D. Macarulla T. Pazo Cid R. Chandana S. R. De La Fouchardière C. (2023). NALIRIFOX *versus* nab-paclitaxel and gemcitabine in treatment-naive patients with metastatic pancreatic ductal adenocarcinoma (NAPOLI 3): a randomised, open-label, phase 3 trial. Lancet London, Engl. 402, 1272–1281. 10.1016/s0140-6736(23)01366-1 37708904 PMC11664154

[B24] Wang J. Taylor A. Showeil R. Trivedi P. Horimoto Y. Bagwan I. (2014). Expression profiling and significance of VEGF-A, VEGFR2, VEGFR3 and related proteins in endometrial carcinoma. Cytokine 68 (2), 94–100. 10.1016/j.cyto.2014.04.005 24845798

[B25] Wattenberg M. M. Asch D. Yu S. O'Dwyer P. J. Domchek S. M. Nathanson K. L. (2020). Platinum response characteristics of patients with pancreatic ductal adenocarcinoma and a germline BRCA1, BRCA2 or PALB2 mutation. Br. J. cancer 122 (3), 333–339. 10.1038/s41416-019-0582-7 31787751 PMC7000723

[B26] Wei M. Liu R. Xu Y. Chen X. Liu C. Bai X. (2024). Phase 1b study of first-line fuzuloparib combined with modified FOLFIRINOX followed by fuzuloparib maintenance monotherapy in pancreatic adenocarcinoma. BMC Med. 22 (1), 365. 10.1186/s12916-024-03581-y 39232761 PMC11375820

