# Supplementary material for: Continuous response to maintenance fuzuloparib for germline BRCA2- mutated metastatic pancreatic adenocarcinoma: a case report and literature review
Source: Front Pharmacol. 2025 Sep 9;16:1656670. doi: 10.3389/fphar.2025.1656670 (PMC12455208; doi:10.3389/fphar.2025.1656670)
Supplement: Supplementary file 1 [file DataSheet1.pdf]

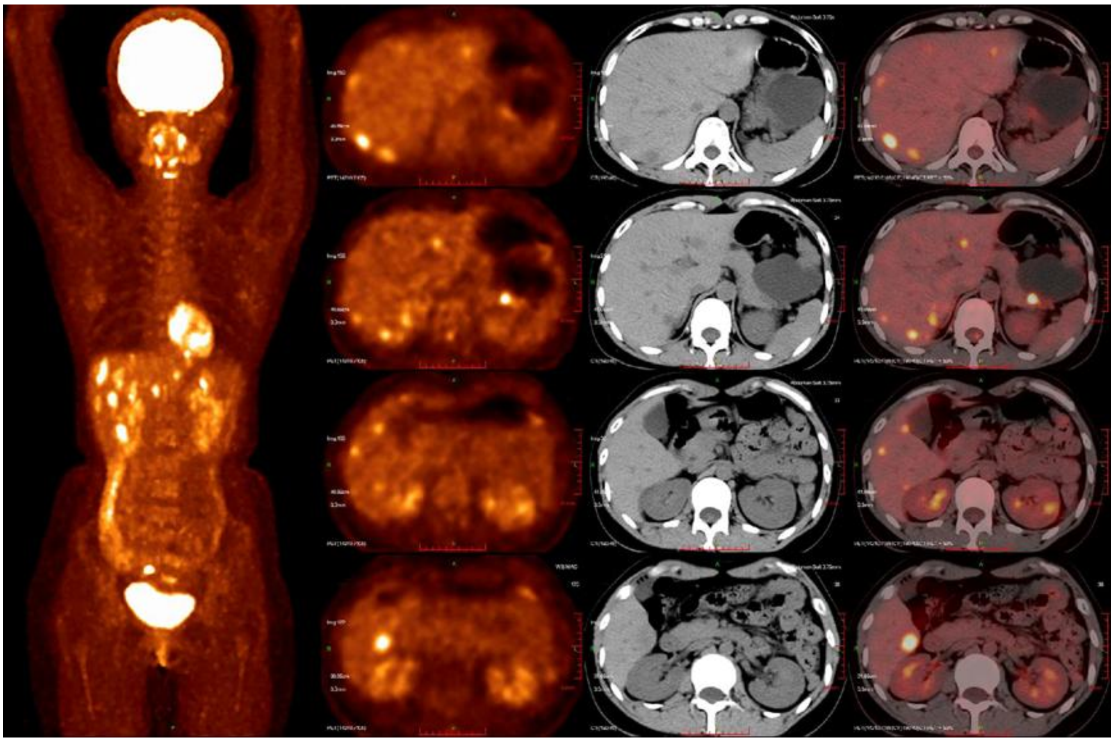

Appendix Figure 1. Baseline PET-CT imaging showing pancreatic ductal adenocarcinoma measuring 66c 62 mm with intense glucose uptake (SUVmax: 8.69) with extensive liver metastases (SUVmax 10.48).
